# Acute Transverse Myelitis after COVID-19 Vaccination

**DOI:** 10.3390/medicina57101010

**Published:** 2021-09-25

**Authors:** Yu-Ting Hsiao, Ming-Jen Tsai, Ying-Hao Chen, Chi-Feng Hsu

**Affiliations:** 1 Department of Emergency Medicine, Ditmanson Medical Foundation Chiayi Christian Hospital, Chiayi City 600, Taiwan; amy810129@gmail.com (Y.-T.H.); tshi33@gmail.com (M.-J.T.); 2 Department of Neurology, Ditmanson Medical Foundation Chiayi Christian Hospital, Chiayi City 600, Taiwan

**Keywords:** transverse myelitis, COVID-19, vaccine

## Abstract

The adverse effects of the COVID-19 vaccine have been discovered as the rapid application of the vaccines continues. Neurological complications such as transverse myelitis raise concerns as cases were observed in clinical trials. Transverse myelitis is a rare immune-mediated disease with spinal cord neural injury, resulting in neurological deficits in the motor, sensory, and autonomic system. Vaccine-related transverse myelitis is even rarer. We present a case of acute transverse myelitis after vaccination against COVID-19 with the ChAdOx1 nCOV-19 vaccine (AZD1222), which was the first case reported in Taiwan. Although it rarely occurs, post-vaccination neurological complications should not be ignored. As the pandemic of SARS-CoV-2 continues to spread and concern about vaccination efficacy and safety rises, heterologous vaccination were implemented in health public policy in several countries. A literature review of several clinical trials shows promising effects of mix-and-match vaccination. Further study on different combinations of vaccines can be expected.

## 1. Introduction

Since the rapid administration of COVID-19 vaccines, specialists are facing questions regarding potential neurological complications. Acute transverse myelitis (ATM) post-vaccination raises concerns as three cases were observed during ChAdOx1 nCOV-19 vaccine (AZD1222) clinical trials among 11,636 participants [1]. Vaccine related ATM is rare. The incidence is approximately 1.739/per million people as reported by the previous COVID-19 vaccine adverse event database [2]. Early diagnosis and treatment could benefit the recovery and prevent reccurrence. The adverse events also raise discussions regarding heterologous vaccination, which has been performed in multiple countries. Here, we present the first case of acute transverse myelitis after vaccination against COVID-19 with AZD1222 in Taiwan and review the related policy regarding adverse events after vaccination from the Taiwan Centers of Disease Control.

## 2. Case Report

A 41-year-old man with diabetes under well medical control who works as an emergency physician received his first dose of AZD1222 2 weeks prior to the onset of symptoms. He first presented with left peripheral facial palsy, which was resolved after oral prednisolone. In the following week, a tingling sensation over T4 dermatome was experienced, followed by progressive paresthesia below T4, along with lower-limb weakness and clumsiness, which developed in the 6th week after vaccination. Neurologic examination revealed the loss of pinprick sensation below T4 bilaterally; decreased lower-limb muscle power, which was more severe over the left side; loss of joint position; and vibration over both lower limbs as well as increased bilateral knee reflex.

**Figure 1:**
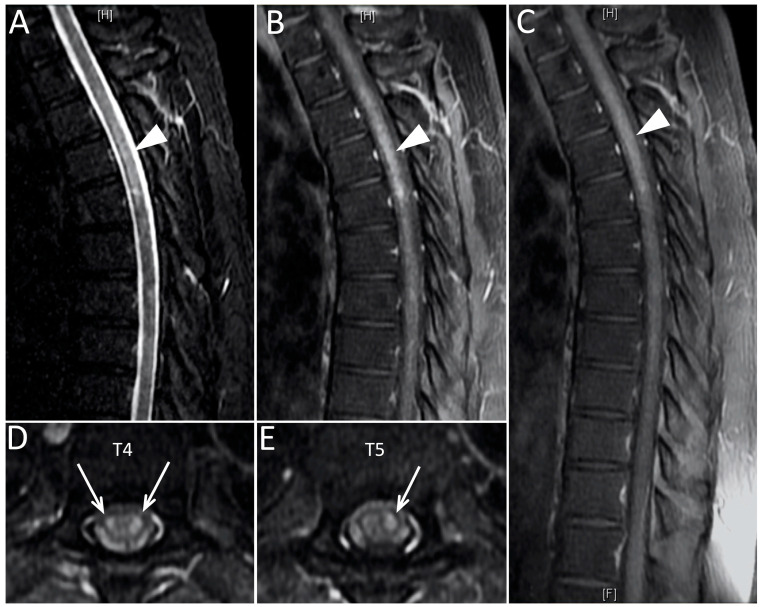
The MRI of the thoracic spine on the T2-weighted short tau inversion recovery scan (**A**) showed a hyperintense lesion on T1-T6 (indicated by arrowheads). The lesion was enhanced in the early-phase contrast-enhanced T1-weighted fast spin echo scan (**B**) and post-contrast T1-weighted fast spin echo scan (**C**). The axial view of the post-contrast T1-weighted fast spin echo scan over T4 (**D**) and T5 (**E**) revealed the enhanced lesion in the spinal cord (indicated by arrows).

**Figure 2:**
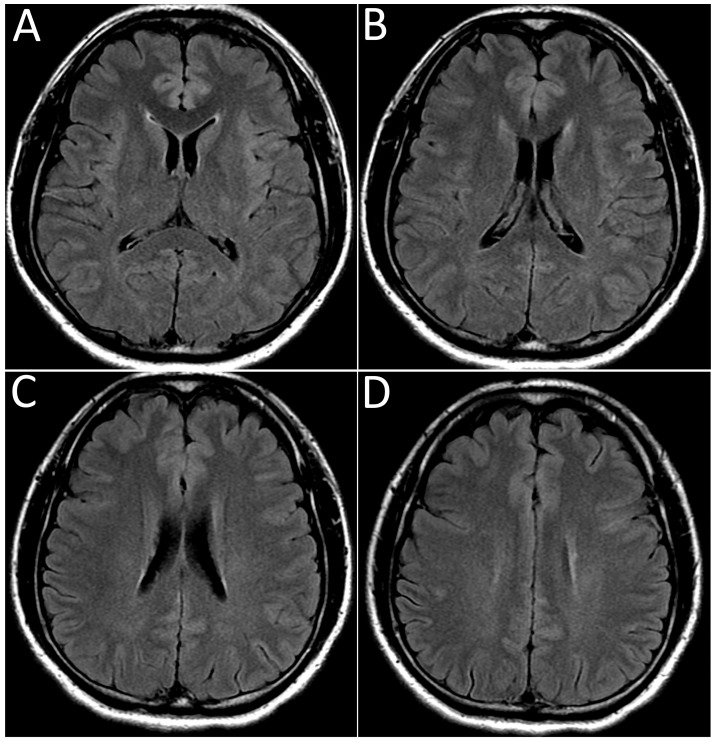
The serial axial MRI (**A**–**D**) of the brain on T2-weighted fluid-attenuated inversion recovery images showed no multiple sclerosis lesion in the periventricular area.

**Figure 3:**
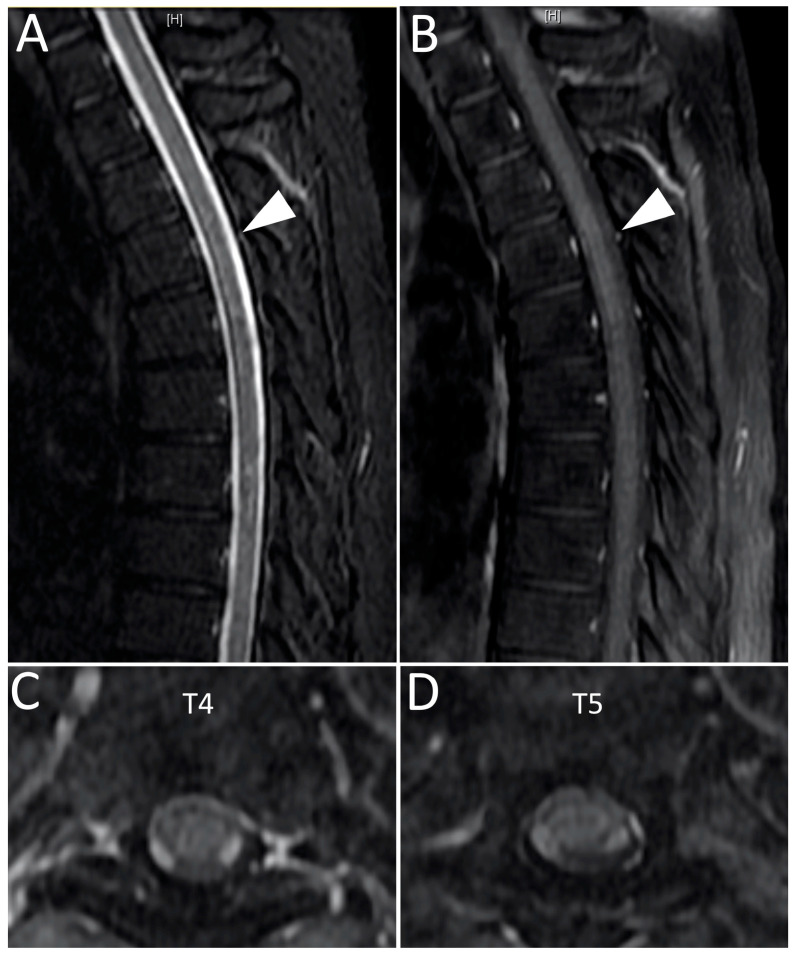
The follow-up MRI 16 weeks after vaccination of the thoracic spine on the T2-weighted short tau inversion recovery scan (**A**) and post-contrast T1-weighter fast spin echo scan (**B**) revealed the recovery of the previous lesion over T1-T6 (indicated by arrowheads). The axial view of the post-contrast T1-weighter fast spin echo scan over T4 (**C**) and T5 (**D**) also had similar findings.

The patient was admitted to the neurology ward for workups 7 weeks post-vaccination. Laboratory analysis showed unremarkable findings in the complete blood cell count, complete metabolic profile, and negative SARS-CoV-2 PCR test. Brain and whole spine magnetic resonance imaging (MRI) were performed. The contrast-enhanced MRI of the spine revealed an intramedullary-enhancing lesion over the spinal cord at the T1 to T6 vertebral levels (Figure 1). The MRI of the brain was normal (Figure 2). Cerebrospinal fluid (CSF) analysis showed mild pleocytosis (WBC:11/uL, lymphocyte predomminant: 100%) and mild elevated protein levels (44.3 mg/dL). Infectious disease of central nervous system was ruled out by the CSF analysis and virus culture. The tumor marker and autoimmune profiles, including the serum rheumatoid factor, serum complement C3 and C4, anti-Ro/La antibody, antinuclear antibody, and anti-ds-DNA antibody, were all in the normal range. Due to longitudinal transverse myelitis, Aquaporin4 antibodies were checked and showed negative. Therefore, neuromyelitis optica was not favored. Multiple sclerosis was also less likely due to the longitudinal transverse myelitis, normal brain MRI, and considering the fact that it could not fulfill the McDonald criteria. Coagulation profiles were also checked with the concern of vaccine-induced immune thrombotic thrombocytopenia, which were all within normal limits. By the typical findings of the spine MRI and clinical symptoms, the patient was finally diagnosed with acute transverse myelitis (ATM) with suspicion of vaccine-associated neurological adverse effect. The patient received pulse therapy with 1000 mg of methylprednisolone daily for 5 days and had a dramatic improvement in the limb weakness. He maintained oral prednisolone with a dose of 1 mg/kg/day for other residual symptoms and was tapered as symptoms gradually subsided. Due to the patient having a major adverse event with AZD1222, the second dose of the vaccine was switched to the Moderna mRNA-1273 vaccine in the 14th week after the first dose and no major adverse event was observed until now. Sixteen weeks after first vaccination, a follow-up contrast-enhanced MRI over the thoracic spine was arranged, which revealed the resolution of the T1-T6 lesion (Figure 3). The patient is now doing well under regular follow-up without neurological sequelae.

## 3. Discussion

Acute transverse myelitis is a rare clinical syndrome in which immune-mediated processes cause spinal cord neural injury, resulting in motor, sensory, and autonomic dysfunction [3]. Since the pandemic of COVID-19 developed, COVID-19-related transverse myelitis has been reported and the incidence is about 0.5 case/per million people, which could account for 1.2% of all COVID-19-related neurological complications [1]. The incidence of ATM is estimated to be up to 3–5 cases per million people a year and vaccine-associated ATM cases are even more rare [3]. According to the Vaccine Adverse Event Reporting System (VAERS) of the United States, a total of 119 post-vaccination ATM cases were reported during the period of 1985 to 2017 [4]. For COVID-19 vaccine-related events, a total of 51,755,447 doses of COVID-19 vaccines were administered until March 2021 and nine cases of related ATM were reported among 9442 adverse events; the incidence is approximately 1.739/per million people [2]. After the literature review, we summarized the published case reports of SARS-CoV-2 vaccination-related transverse myelitis with detailed clinical information in Table 1 [5,6,7,8,9,10,11,12].

According to the VAERS of the Taiwan Centers for Disease Control, since the beginning of vaccination administration on 22 March 2021 to 28 July 2021, there were 9,987,157 doses of vaccines administered in Taiwan and 5620 adverse effect cases reported. Among the 2548 cases of severe adverse events, neurological complications, including 208 strokes, 42 facial palsy, seven Guillain–Barre Syndrome reports, two acute disseminated encephalomyelitis, and two transverse myelitis, were recognized [13]. This is the first case reported of acute transverse myelitis after COVID-19 vaccination in Taiwan.

The pathogenesis of transverse myelitis is thought to be immune-mediated from infection, para-infection, autoimmune disease, and paraneoplastic [6]. Related pathogens include cytomegalovirus, varicella-zoster virus, Epstein bar virus, and coxsackieviruses [14]. The proposed mechanism of the post-infection neurological disorder is the concept of “Molecular Mimicry”, which means that the microorganism epitope shares a similar structure to the host’s antigen. The cross-reaction between the epitope and self-antigen activates B lymphocyte and the bystander activation of T cells, which induces the immune response. These mechanism appears to be the explanation for the vaccine with viral antigen adjuvants, which might mediate immune responses targeting the spinal cords [1,6,7,15]. This could be somehow explained by the pleocytosis found in patients’ CSF considering that the blood–brain barrier might have been broken down within a focal area of the spinal cord [6,15]. It is noticeable that both the AZD1222 and Johnson & Johnson COVID-19 vaccines contain adenovirus antigens, and they might induce ATM by the same pathogenesis [1,7]. As other vaccines are without a viral vector, a similar hypothesis was proposed that immune dysregulation secondary to vaccination might trigger ATM [6]. However, the clear causal relation between the SARS-CoV-2 vaccine and ATM is still an issue for further investigation.

Due to the concerns regarding vaccine adverse effects and the persisting pandemic of COVID-19, heterologous vaccine regimens raise discussions. Some countries are now performing heterologous prime-boost vaccine schedules [16]. Currently in Taiwan, people who developed major adverse events after the first dose of vaccination are strongly suggested to receive the second dose vaccine with a different mechanism. Clinical trials regarding the safety and efficacy have been conducted. Heterologous vaccination with either the adenoviral-vectored or mRNA vaccine induced a higher immune response than that of homologous vaccinations in mice [17]. An interim analysis in the UK Com-COV trial, which investigated heterologous prime-boost regimens of the AZD1222/BNT162b2 COVID-19 vaccine, found increased reactogenicity following heterologous boost with BNT162b2 rather than with homologous AZD1222 after the initial vaccination with AZD1222 in a 28-day interval, but with greater systemic reactogenicity after the boost [16]. In a German study comparing the prime-boost vaccine schedule of BNT162b2/BNT162b2 and AZD1222/BNT162b2, T-cell reactivity was significantly higher after heterologous AZD1222/BNT162b2 boost immunization compared to homologous BNT162b2/BNT162b2 boost immunization [18]. A small cohort study in Sweden also reported similar results [19]. Studies regarding the immune response against different virus strains also found advantages in heterologous groups [19,20]. Other studies are evaluating the effects of the heterologous prime-boost on the mRNA-1273 vaccine (Moderna). Promising results are expected [16].

## 4. Conclusions

Although they rarely occur, the association of the COVID-19 vaccine and the disease, along with other neurological complications, should not be ignored. As mix-and-match COVID-19 vaccines are under greater discussion in Taiwan, studies examining it are also on the way. As I (C.-F.H., the corresponding author) am the patient described above, I recommend that clinicians keep vaccine-related neurological complications in mind. Delayed diagnosis and treatment may cause sequela. I also propose that my experience of the heterologous vaccination could open up discussion regarding the control of the COVID-19 pandemic.

## Figures and Tables

**Table 1:** Summary of the published case reports of SARS-CoV-2 vaccination-induced transverse myelitis in the literature.

Reference	Age and Gender	Type of Vaccine	Onset Time *	Clinical Presentations	Involved Region	Management	Outcome
Notghi et al. [5]	58 Male	AstraZeneca	7 days	Lower-limb numbness, genital dysesthesia, urinary incontinence, and hyperreflexia	T2–T10	IV methylprednisolone then oral prednisolone Plasmapheresis	Improved
Khan et al. [6]	67 Female	Moderna	1 day	Four limbs’ weakness and hyperreflexia, and loss of vibration up to ankle	C1–C3	IV Methylprednisolone and plasmapheresis	Improved
Tahir et al. [7]	44 Female	Johnson & Johnson	7 days	Back pain, urinary retention, paresthesia in neck and abdomen, numbness, weakness, and hyperreflexia in the lower extremities	C2–C3 and T2	IV prednisolone and plasmapheresis	Improved
Erdem et al. [8]	78 Female	CoronaVAC	3 weeks	Tetraparesis, urinary retention, and paresthesia of bilateral upper extremities	C1–T3	No information	No information
Pagenkopf et al. [9]	45 Male	AstraZeneca	1 week	Thoracic back pain, urinary retention, acute flaccid tetraparesis, and sensory level at T9	C3–T2	IV prednisolone	Improved
Helmchen et al. [10]	40 Female	AstraZeneca	2 weeks	Back pain, incontinence, paraplegia, and paresthesia under abdomen level	T7–T10 and optic neuritis	IV Methylprednisolone, plasmapheresis, and immunoadsorption	Improved
Singh et al. [11]	36 Male	AstraZeneca	8 days	Abnormal sensations in both lower limbs and sense of vibration impaired up to sternum	T2	IV Methylprednisolone	Improved
Vegezzi et al. [12]	44 Female	AstraZeneca (ABV 2856)	4 days	Bilateral plantar feet ascending paresthesia and reduced sensation in lower back	T7–T8 and T10–T11	IV Methylprednisolone	Improved
This case report	41 Male	AstraZeneca	2 weeks	Paresthesia below T4, lower-limb weakness and clumsiness, loss of joint position and vibration, and hyperreflexia	T1–T6	IV Methylprednisolone and then oral prednisolone	Improved

* Onset time refers to the time duration from vaccination to symptom onset.

## Data Availability

The data that support the findings of this paper are available from the corresponding author, C.-F.H., upon reasonable request.
